# The impact of unmet treatment need on oral health related quality of life: a questionnaire survey

**DOI:** 10.1186/s12903-024-04169-x

**Published:** 2024-04-08

**Authors:** Akshata Shetty, Rahul Bhandary, Dhruv Ahuja, Geetu Venugopalan, Enzo Grossi, Guilia Margherita Tartaglia, Shahnawaz Khijmatgar

**Affiliations:** 1 Nitte (Deemed to be University), Department of Periodontics, A B Shetty Institute of Dental Sciences, Mangalore, Karnataka India; 2 Department of Orthodontics and Dentofacial Orthopedics, Manav Rachna International Institute of Research and Studies (MRIIRS), Manav Rachna Dental College, Faridabad, Haryana India; 3 Nitte (Deemed to be University), Department of Oral Biology and Genomic Studies, A B Shetty Memorial Institute of Dental Sciences, Mangalore, Karnataka India; 4 Villa Santa Maria Institute, Tavernerio, Italy; 5 School of Medicine, University of Madrid, Madrid, Spain; 6 SC Chirurgia Maxillo-Facciale e Odontostomatologia, Fondazione IRCCS Ca’ Granda Ospedale Maggiore Policlinico, Milano, Italy

**Keywords:** Health, Oral, Dental, Delay, Treatment, Policies, Gingivitis, Lesion, Carious, Psychology, Social, OHIP-14

## Abstract

**Background:**

Based on the present global burden of oral diseases, unmet dental needs affect a more significant population worldwide. It is characterised by the need for dental care but receiving delayed or no care. The contributing factors include lack of knowledge about oral health, its consequences, and the availability of dental services. We need to find out the scale of the problem of unmet dental needs for the south Indian population. Therefore, the objective was to determine the relationship between the presence of oral disease and the quality of life-related to oral health using the OHIP-14 tool.

**Methods:**

The unmet dental requirements of the south Indian population were determined using a cross-sectional questionnaire survey. Close-ended questions were used to obtain data from two investigators trained to record the answers from the patients. The data was collected using the OHIP-14 questionnaire, which consists of 14 items divided into seven domains with two questions each. Physical pain, psychological impairment, physical disability, psychological disability, social disability, and disability were all considered. An additional analysis of artificial neural network (ANN) was done.

**Results:**

The response rate was 100 per cent. *N* = 1029 people replied to the questionnaire about their unmet dental needs. *N* = 497 (48.3%) were men, whereas *N* = 532 (51.7%) were women. The average age was 31.7811.72. As their current occupation, most of the included subjects (60.1%) were students. The respondents had no known personal habits and a mixed diet (94.93%). The average BMI was 24.022.59 (14-30.9). OHIP was present in 62.3% of the population. The average OHIP-14 severity score was 10.97. (8.54). The severity and degree of unmet dental need were substantial (p0.01) due to pain in the mouth/teeth/gums, malocclusion, and gum bleeding. The most common OHIP-14 domains affected by unmet oral needs were psychological discomfort, psychological limitation, social limitation, and feeling handicapped. The analysis of ANN revealed that high OHIP scores were primarily attributed to dental caries, poor oral health, and dental aesthetics.

**Conclusion:**

The severity and degree of unmet dental needs were significant among the south Indian population. The most common oral health status that impacted OHIP-14 domains were pain, malocclusion, and bleeding gums. These patients were significantly impacted by psychological discomfort and social limitations and felt handicapped.

## Introduction

Oral health is a good predictor of overall health. The link between oral and general health has only recently been established. However, both healthcare experts and the general public have ignored this evidence. The issue with healthcare professionals is a lack of trust in research and the belief that research cannot be translated into clinical practice. Another issue is that health institutions have failed to address the problems of oral and general health. One cause could be a considerable gap between policymakers, researchers, health professionals, and the general public. Decisions are made without consulting with several stakeholders. Most research funding themes and calls are advertised with one main objective, i.e. to make the world a better place to live through innovation and better healthcare services. However, given the magnitude of the problem (Global Burden of Disease 2017 estimated that oral disease affects 3.5 billion people), this goal still needs to be met. The idea that oral disorders cause a significant health burden and influence quality of life, finances, discomfort, and even mortality should be accepted by all stakeholders. The issue was recently well addressed by Peres MA (2019); Watt RG (2019); The LC (2019); Moynihan P (2020); Watt RG (2020) [1–6].

One possible solution to the abovementioned issue is to conduct research and formulate a specific research topic relating to unmet requirements in oral health care. The unmet health care needs variables that are used to measure equity in access to health care services. They refer to the proportion of people aged 15 and up who thought they required health treatment in the previous 12 months but did not access due to financial constraints, long waiting lists, or transportation issues. In 2018, 4% of the EU population lacked access to dental care [7]. The Indian population’s unmet dental needs are widespread, accounting for 62% of the total [8–10]. However, this must be examined concisely through well-designed studies.

The oral health related quality of life (OHRQOL) is a multidimensional term capturing people’s perception of significant factors in their present-day life [11]. Slade and spencer introduced the OHIP formula in 1994, and the metric measurements were function, pain, physical disability, social disability, and disability. These are social metrics used to measure the impact of oral conditions at the level of society that will be relevant to policymakers [12]. Allison J et al. claimed that understanding the importance of the social consequences of diseases and that medical interventions are intended to increase the length and quality of life are two driving factors that change how we think about health and health care. For these purposes, the quality, efficacy, and healthcare performance are often judged by their effect on the “quality of life” of a patient [13].

As a result, it is critical to understand that measurements of quality of life are not a replacement for calculating illness outcomes but rather a supplement to them. Because dentistry has stayed strictly scientific in its approach to oral health, which equates health with illness, the quality of life linked with oral health is a relatively new but quickly rising trend [11].

We hypothesize that there is a significant association between unmet oral healthcare needs and the oral health-related quality of life (OHRQoL) among adults attending outpatient clinics. We expect that individuals with unmet dental treatment needs, such as dental caries, missing teeth, and oral pain, will report a lower OHRQoL compared to those without such needs. We also anticipate that factors like age, education level, and specific oral health conditions will play a crucial role in determining the impact on OHRQoL.

The rationale for this hypothesis is grounded in the existing literature, which suggests that oral health is intricately connected to an individual’s overall well-being. Our study highlights the prevalence of oral health issues, such as dental caries, missing teeth, and pain, and their potential impact on the quality of life. It also identifies specific demographic factors, such as age and education level, as potential influencers of OHRQoL. Given the importance of oral health and the significant burden it places on individuals, it is essential to investigate the relationship between unmet oral healthcare needs and the quality of life experienced by adults attending outpatient clinics. Nevertheless, in our study, we aimed to assess the oral health-related quality of life in adults attending the outpatient clinic and to determine the relationship between the presence of oral disease and the quality of life-related to oral health using the OHIP-14 tool.

### Research question

Is there a significant association between unmet oral healthcare needs, including dental caries, missing teeth, and oral pain, and the oral health-related quality of life (OHRQoL) among adults attending outpatient clinics?

## Materials and methods

A cross-sectional questionnaire study by a random sampling method was conducted among the adult population attending the integrated clinics OPD at A.B Shetty Dental College, Mangalore, after receiving the institutional ethical clearance. The ethical approval number is ABSM/EC8/2019. A written informed consent was obtained from all participant’s for inclusion in the study. The questionnaire used in this study to assess oral health-related quality of life (OHRQoL) underwent a meticulous development process to ensure its relevance and effectiveness. Beginning with an extensive literature review, a pool of potential questionnaire items was generated, covering key dimensions of OHRQoL. These items were subjected to content validity through expert review, where a panel of dental professionals (S.K; R.B; A.S) assessed their alignment with the theoretical framework of OHRQoL. The questionnaire’s clinical relevance and accuracy in measuring OHRQoL were verified by two independent clinicians, Clinician S.K and Clinician A.S, whose inputs helped refine the instrument. The training of two investigators, A.S. and D.A., on collecting and recording the data was carried out. The comprehensive development, content validity, face validity, construct validity, and reliability assessments of the questionnaire ensured its effectiveness as a tool for measuring OHRQoL in the study.

This questionnaire survey was reported according to the checklist of reporting survey studies (CROSS) [14]. The inclusion requirements were patients over 18 with unmet dental treatment, with dental conditions such as dental caries, periodontitis, teeth crowding or missing teeth, and who could understand English or Hindi. The study population were patients attending the outpatient clinic, at A.B. Shetty Dental College, Mangalore, with the above inclusion criteria. The patients with mentally incapacitated and diagnosed pregnancies were excluded from the study. The data collection instrument was the OHIP-14 questionnaire, which consists of 14 questions in seven domains with two questions each. Functional disabilities, physical pain, psychological impairment, physical disability, psychological disability, social disability, and disability were included. The answers to the questions were based on a Likert scale ranging from 0-“never” to 4- “very often” [11]. The questionnaire also contains variables such as (a) demographic variables and (b) dentition status will be checked and registered. C) dental treatment needs due to the occurrence of dental caries, missing teeth, and teeth-related pain will be reported as “present or absent” and deemed “unmet dental treatment needs.” The inter and intra variability was tested for two survey collecting persons using 20 patients and standardized till the 80% score above was achieved. There was no follow-up of the patients after collecting the survey information from the first point of contact.

### Data Management

Total OHIP score for the respondents was calculated by adding the response score for each item to give a minimum score of 0 and a maximum score of 56. An impact on the quality of life was considered at a response level hardly ever. The discriminant validity will be determined by comparing OHIP-14 scores in those with or without treatment needed due to dental caries, periodontitis, missing teeth and pain associated with teeth.

## Statistics

### Sample size calculation

Using the power and sample size estimation STATA software version 17.0 version, *N* = 1046 sample size was determined as 386 based on the prevalence of dental disorders per cent among adults in Mangalore 0.05 (α = 0.05) precision. Therefore, a suitable sample size of *n* = 1000 patients was needed to identify the unmet dental treatment needs and their impact on oral health and quality of life with 80% power, using a two-sample t-test and assuming a (two-sided) α of 0.05 and β = 0.20.

### Data analysis

Data collected will be processed and analyzed with STATA 17.0 version software. Data Analysis Strategies: A association test will be done using a 95% confidence interval, descriptive statistics and, Mann Whitney U test, ANOVA analysis. Internal validity was determined using Cohen Kappa Test. The correlation coefficient was estimated and interpreted as Schober P (2018) described [15].

An artificial adaptive system called Auto-CM was used to graphically show the most important connections among variables (Buscema et al., 2008). Auto-CM is a special kind of ANN that develops weights that are proportional to the strength of the associations of all variables each other. The weights are then transformed in physical distances so that couples of variables whose connection weights are higher become nearer and vice versa. After the training phase, the weights matrix of the Auto-CM represents the warped landscape of the dataset. Subsequently, a minimum spanning tree filter was applied to the weights matrix of the Auto-CM system to obtain a map of the main connections between the variables of the dataset and the basic semantic of their similarities, defined connectivity map as detailed elsewhere (Buscema et al., 2008) [16].

## Results

**Fig. 1:**
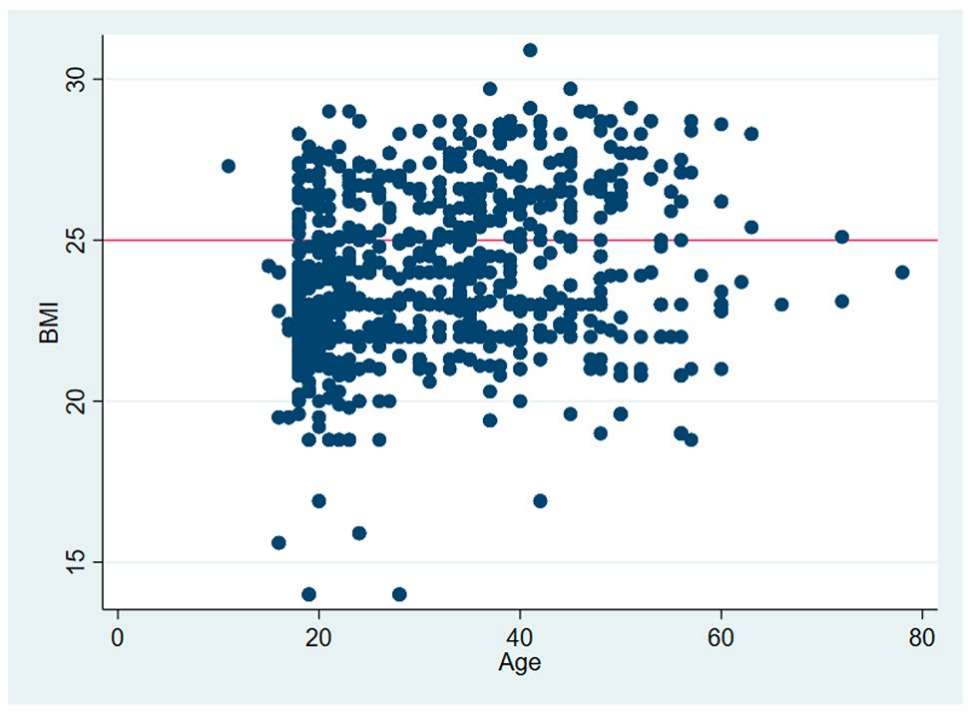
Age V BMI. The orange line representing the cut-off BMI i.e. 24.9 above which a person is considered as over-weight and obese. Nearly, 50% of the included subjects are overweight and obese

**Fig. 2:**
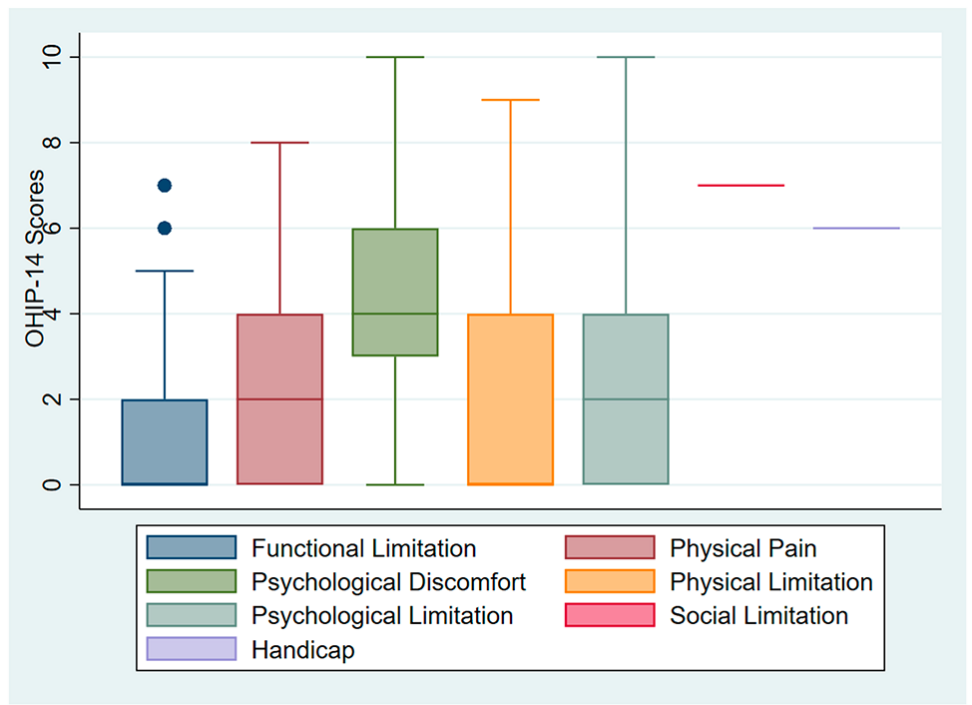
OHIP-14 Scores for Domains

The response rate was 100% and inter-variability among two survey recording was more than 85%. A total of *N* = 1029 subjects responded to the questionnaire regarding their unmet dental needs. *N* = 497 (48.3%) were males, and *N* = 532 (51.7%) were females. The overall mean age was 31.78 ± 11.72. Most included subjects were students (60.1%) in their current occupation (Table 1). The distribution (%) of OHIP-14 was illustrated in Table 2. The mean BMI was 24.02 ± 2.59 (14-30.9) (Fig. 1). The OHIP-14 score was illustrated in Fig. 2 for different domains. Nearly 42.6% had an absence of sound teeth, 42.6% had decayed teeth, 37.8% had missing teeth, 16.5% had pain in the oral cavity due to different reasons, and 70.0% had bleeding gums. OHIP prevalence was present in 62.3% of individuals. The mean OHIP-14 severity was 10.97(8.54) (Table 1). Psychological discomfort has high OHIP-14 scores; by gender, males have higher OHIP-14 scores. The prevalence of oral health impact due to unmet dental needs was more among secondary level education and severe in PUC/Diploma level of education. The severity and extent of unmet dental needs were significant due to pain in the mouth/teeth/gums, malocclusion, and bleeding of gums (Table 3). Psychological discomfort, psychological limitation, social limitation, and feeling handicapped were the most common OHIP-14 domains affected due to unmet dental needs (Table 2).

**Table 1:** Distribution of study population according to sociodemographic factors and unmet dental needs

		n	%
Age (in years)	≤ 24	368	35.8
	25–34	253	24.6
	35–44	236	22.9
	45–54	125	12.1
	≥ 55	47	4.6
Gender	Male	497	48.3
	Female	532	51.7
Education	Less than secondary	118	11.5
	Secondary	618	60.1
	PUC/Diploma	110	10.7
	Graduation – Postgraduation	183	17.8
Location	Karnataka	348	33.8
	Kerala	641	66.2
Sound teeth	Absent	976	94.8
	Present	53	5.2
Decayed teeth	Absent	438	42.6
	Present	591	57.4
Missing teeth	Absent	640	62.2
	Present	389	37.8
Pain in mouth/teeth/gums	Absent	859	83.5
	Present	170	16.5
Malocclusion	Absent	692	67.2
	Present	337	32.8
Bleeding gums	Absent	309	30.0
	Present	720	70.0
Mobility of teeth	Absent	930	90.4
	Present	99	9.6
Fractured teeth	Absent	1021	99.2
	Present	8	0.8
Grossly decayed teeth	Absent	698	67.8
	Present	331	32.2
OHIP prevalence	Absent	388	37.7
	Present	641	62.3
OHIP-14 extent mean (SD)		1.63 (2.13)	
OHIP-14 severity mean (SD)		10.97 (8.54)	

**Table 2:** Distribution (%) of OHIP-14

OHIP-Domain	OHIP-14	Never	Hardly ever	Occasionally	Fairly often	Very often
Functional Limitation	Difficulty in pronouncing words	74.8	11.1	9.2	4.1	0.8
	Worsened sense of taste	74.8	11.4	8.7	3.6	1.5
Physical Pain	Pain in mouth	65.5	7.0	17.2	7.9	2.4
	Uncomfortable in eating any food	51.2	10.6	23.2	10.7	4.3
Psychological Discomfort	Feeling self-conscious	11.6	8.3	29.6	32.8	17.8
	Felt tense	35.6	13.1	26.8	16.9	7.6
Physical Limitation	Unsatisfactory diet	66.6	14.5	18.6	6.2	4.2
	Meals interrupted	69.0	13.6	9.6	5.2	2.6
Psychological Limitation	Difficulty in relaxing	66.3	17.5	11.3	3.7	1.3
	Feeling embarrassed	47.8	18.3	20.6	7.6	5.7
Social Limitation	Irritation with other people	70.6	15.9	19.4	3.5	0.6
	Difficulty to do usual jobs	77.1	12.1	7.7	2.9	0.3
Handicap	Life was less satisfactory	72.7	9.7	9.7	5.2	2.7
	Not able to function totally	91.8	4.5	2.7	0.7	0.3

**Table 3:** Distribution of study population according to sociodemographic factors and unmet dental need by prevalence, extent and severity of OHIP-14

		n	Prevalence of ≥ 1 oral health impacts (*n* = 641) %	*p*	Extent of oral health impacts µ (± SD)	*p*	Severity of oral health impacts µ (± SD)	*p*
Age (years)	≤ 24	368	66.6	0.102	1.59 (1.95)	0.828	10.30 (7.87)	0.010**
	25–34	253	63.6		1.62 (2.25)		11.13 (8.91)	
	35–44	236	55.9		1.51 (2.00)		10.40 (8.01)	
	45–54	125	60.8		1.70 (2.37)		12.58 (9.62)	
	≥ 55	47	57.4		1.87 (2.60)		13.85 (10.12)	
Gender	Male	532	60.0	0.110	1.63 (2.23)	1.000	11.11 (8.93)	0.591
	Female	497	64.8		1.63 (2.02)		10.82 (8.11)	
Education	Less than secondary	118	50.8	0.008*	1.54 (2.51)	0.440	12.14 (9.22)	0.049**
	Secondary level	618	61.7		1.65 (2.15)		10.98 (8.55)	
	PUC/Diploma	110	65.5		1.37 (1.79)		9.08 (7.39)	
	Graduate-Post graduate	183	69.9		1.78 (1.95)		11.29 (8.59)	
Location	Karnataka	348	63.8	0.478	1.83 (2.15)	0.031**	11.55 (8.38)	0.118
	Kerala	641	61.5		1.53 (2.11)		10.67 (8.61)	
Sound teeth	Absent	976	63.2	0.009*	1.63 (2.11)	0.933	10.95 (8.49)	0.757
	Present	53	45.3		1.61 (2.48)		11.32 (9.44)	
Decayed teeth	Absent	438	65.5	0.066	1.75 (2.24)	0.103	11.32 (9.16)	0.254
	Present	591	59.9		1.54 (2.04)		10.71 (8.05)	
Missing teeth	Absent	640	60.6	0.157	1.53 (2.01)	0.047**	10.39 (8.40)	0.005**
	Present	389	65.0		1.80 (2.30)		11.92 (8.69)	
Pain in mouth/teeth/gums	Absent	859	59.5	< 0.001*	1.46 (2.03)	< 0.001**	10.07 (8.30)	< 0.001**
	Present	170	76.5		2.47 (2.40)		15.52 (8.29)	
Malocclusion	Absent	692	58.4	< 0.001*	1.53 (2.07)	0.033**	11.08 (8.59)	0.550
	Present	337	70.3		1.83 (2.23)		10.74 (8.44)	
Bleeding gums	Absent	309	72.2	< 0.001*	2.21 (2.47)	< 0.001**	13.48 (9.40)	< 0.001**
	Present	720	58.1		1.38 (1.91)		9.89 (7.91)	
Mobility of teeth	Absent	930	61.9	< 0.468	1.67 (2.18)	0.052	11.04 (8.75)	0.388
	Present	99	65.7		1.23 (1.48)		10.26 (6.21)	
Fractured teeth	Absent	1021	62.1	0.140	1.62 (2.13)	0.183	10.95 (8.56)	0.424
	Present	8	87.5		2.63 (1.60)		13.38 (5.95)	
Grossly decayed teeth	Absent	698	63.8	0.161	1.71 (2.16)	0.070	11.20 (8.625)	0.203
	Present	331	59.2		1.45 (2.06)		10.47 (8.35)	

*p value < 0.05 estimated using Chi-squared test; ** *p* value < 0.05 estimated using one-way ANOVA.

The domains of OHIP-14 and personal dental unmet needs were compared to demonstrate a linear link between the two variables (Tables 3 and 4). There was a moderately favourable relationship between dental caries and functional limitation and a substantial relationship between poor oral health, physical pain, and psychological discomfort (Fig. 2) (Table 4). Furthermore, there was a significant association between dental caries and psychological restriction.

**Table 4:** Factors associated with prevalence, extent and severity of OHIP-14

		Prevalence of oral health impacts OR (95% CI)^a^	Extent of oral health impacts β (95% CI)^b^	Severity of oral health impacts β (95% CI)^b^
Intercept			2.33 (1.65–3.01)	16.24(13.27–19.21)
Age (in years)	≤ 24	1.37 (0.72–2.62)	-0.41 (0.32–1.04)	-3.93 (-6.39- -1.48)*
	25–34	1.28 (0.66–2.49)	-0.36 (-1.00-0.28)	-2.77 (-5.28- -0.25)*
	35–44	1.07 (0.55–2.06)	-0.31 (-0.96-0.34)	-3.42 (-5.95- -0.88)*
	45–54	1.23 (0.60–2.50)	-0.28 (-0.97-0.41)	-1.79 (-4.52- 0.94)*
	≥ 55	Ref	Ref	Ref
Gender	Male	0.88 (0.67–1.15)	0.11 (-0.15-0.36)	0.43 (-0.57-1.44)
	Female	Ref	Ref	Ref
Education	Less than secondary	Ref	-----	Ref
	Secondary level	0.50 (0.30–0.82)*	-----	-1.04 (-2.63- 0.55)
	PUC/Diploma	0.71 (0.49–1.03)	-----	-3.57 (-5.66- -1.48)*
	Graduate-Post graduate	0.76 (0.45–1.29)	-----	-1.36 (-3.23- 0.51)
Location	Karnataka	-----	Ref	Ref
	Kerala	-----	-0.17 (-0.44-0.10)	-0.39 (-1.46- 0.69)
Sound teeth	Absent	Ref	-----	-----
	Present	0.64 (0.36–1.16)	-----	-----
Decayed teeth	Absent	Ref	Ref	-----
	Present	0.82 (0.62–1.07)	-0.24(-0.50-0.02)	-----
Missing teeth	Absent	Ref	Ref	Ref
	Present	1.34 (1.01–1.77)*	0.38 (0.11–0.64)*	1.45 (0.43–2.47)*
Pain in mouth/teeth/gums	Absent	Ref	Ref	ref
	Present	2.39 (1.60–3.56)*	1.07 (0.72–1.42)*	5.25 (3.92–6.59)*
Malocclusion	Absent	Ref	Ref	-----
	Present	1.60 (1.17–2.18)*	0.37 (0.08–0.66)*	-----
Bleeding gums	Absent	Ref	Ref	Ref
	Present	0.63 (0.46–0.85)*	-0.68 (-0.96- -0.39)*	-3.32 (-4.41- -2.22)*
Mobility of teeth	Absent	Ref	Ref	-----
	Present		-0.40 (-0.83-0.04)	-----
Fractured teeth	Absent	Ref	Ref	-----
	Present	5.15 (0.59–45.41)	1.03 (-0.41-2.47)	-----
Grossly decayed teeth	Absent	Ref	Ref	-----
	Present	0.83 (0.62–1.11)	-0.31 (-0.58- -0.04)*	-----

^a^: Multilogistic regression model; ^b^: Generalized linear model; * *p* value < 0.05 and is statistically significant; OR = Odds ratio; CI = Confidence interval; -----not included in the model

**Fig. 3:**
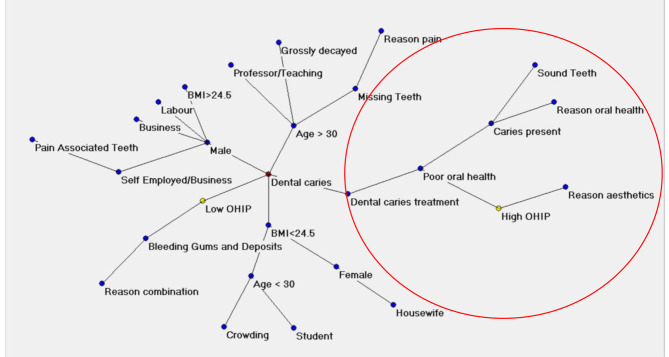
Artificial Neural Network (ANN)

**Fig. 4:**
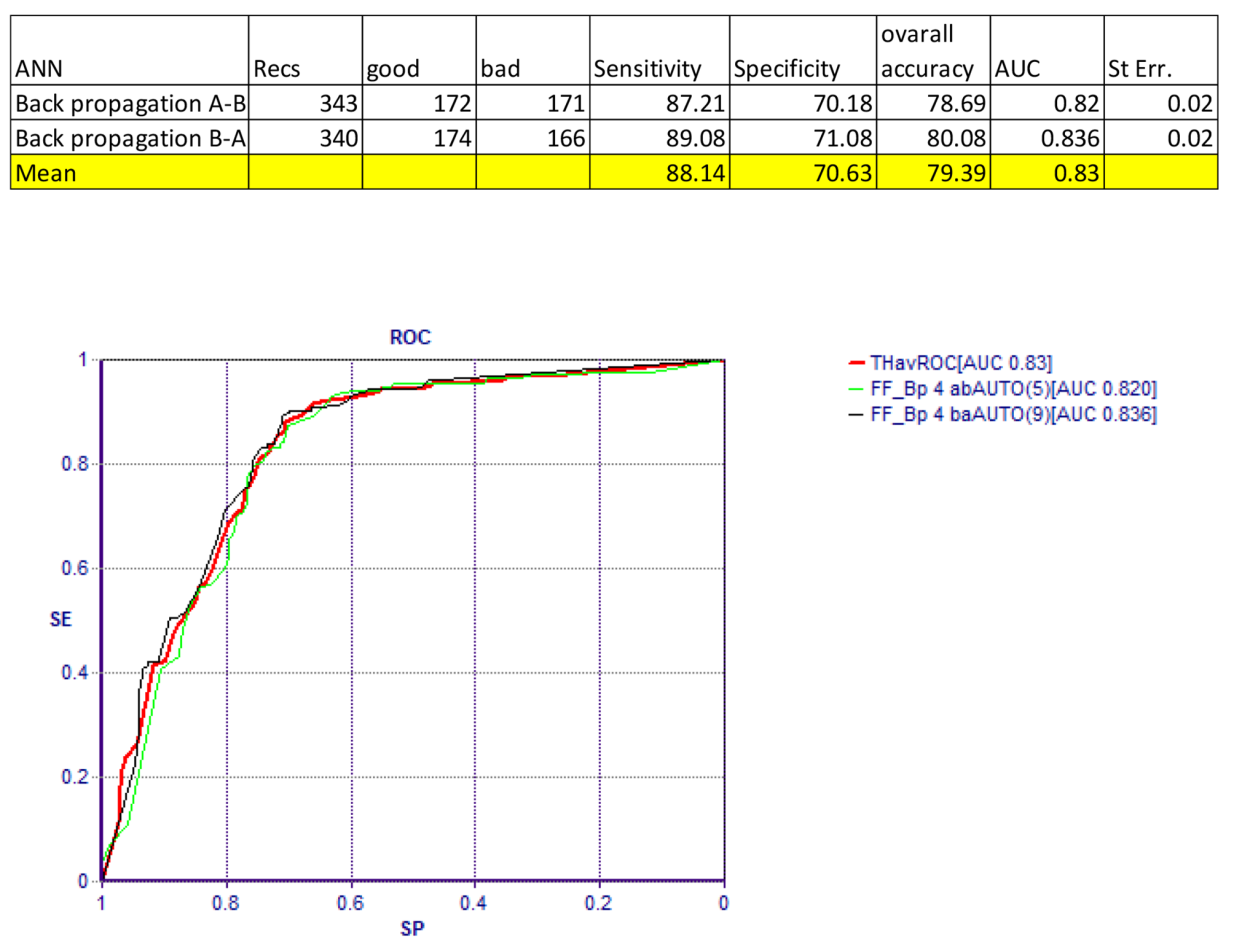
ROC curve. Predicting good(< 7) of bad(> 16) OHIP Score

The analysis of the Artificial Neural Network (ANN) revealed that high OHIP (Oral Health Impact Profile) scores were primarily attributed to dental caries, poor oral health, and dental aesthetics. In other words, these factors emerged as common predictors for elevated OHIP scores. This suggests that dental caries, poor oral health, and issues related to dental aesthetics play a significant role in influencing oral health-related quality of life, as indicated by OHIP scores (Figs. 3 and 4).

## Discussion

When OHRQoL assessments are combined with traditional clinical procedures for assessing oral health status, a more comprehensive assessment of the impact of oral problems on the various dimensions of subjective well-being is achievable [10]. OHIP-14 is a 14-item questionnaire to assess self-reported functional limitation, discomfort, and impairment caused by oral diseases [9]. It is based on an initial extended version of 49 items developed by the WHO and customized for oral health by Locker [11]. In this approach, the effects of oral disease are related from a biological level (impairment) to a behavioural level (functional limitation, discomfort, and disability), and lastly, to a social level (handicap). Despite being a brief questionnaire, the OHIP-14 has been shown to have acceptable reliability and sensitivity and adequate cross-cultural consistency. It is one of the most widely used OHRQoL indicators worldwide and is available in various languages [12].

Majority of the sample belonged to young age group and were pursuing education. Most of them were unemployed and complained to not having sound teeth (94.8%). Our study results found that, older age group ( > = 55 years), lower levels of education, missing teeth, pain, and the presence of bleeding gums had the feeling of being self-conscious and tensed were more commonly reported than any other impacts. Recent studies highlight the significant burden placed on older adults following facial trauma, extending beyond the physical injury itself. While dental, dentoskeletal malocclusion, and maxillofacial trauma in this population present distinct needs, a common thread emerges: the potential for major emotional, social, and physical consequences [17–19].

Absence of sound teeth, decayed teeth, missing teeth, pain in the oral cavity due to different reasons and bleeding gums were associated with the greater extent of OHIP-14. The results identified of an individual’s perception of oral health and its relevance and impact on their life in our study are similar to the study done by Echeverria MS (2018) [20]. Due to these factors, there was psychological discomfort, psychological limitation, social limitation and feeling handicapped (91.8%) among the most common OHIP-14 domains affected due to unmet dental needs in our study.

Artificial Neural Network (ANN) uncovered that elevated OHIP (Oral Health Impact Profile) scores were predominantly associated with dental caries, suboptimal oral health, and concerns regarding dental aesthetics. In essence, these factors emerged as prevalent indicators linked to higher OHIP scores. This implies that dental caries, subpar oral health, and matters related to dental aesthetics significantly impact the quality of life concerning oral health, as signified by OHIP scores (Figs. 3 and 4).

Echeverria MS (2018) [20] found that an increase in the OHIP-14 score among 40.6% of older persons with lower levels of education caused considerable psychological distress owing to tooth loss and pain in teeth. The fact that education is the primary mediator between socioeconomic level and health status explains this finding. The current study’s findings support previous cross-sectional research, which found that older persons with fewer teeth have a more considerable influence on OHRQoL than those with more teeth [3, 13, 21].

Given that our study participants were students, it is still being determined how much oral health literacy they got during their education. Currently, there is considerable interest in evaluating the impact of tooth loss, as it directly influences the quality of life in this age group due to negative impacts on chewing, speaking, nutrition, aesthetics, psychological aspects, self-esteem and social relations [22–25]. Thus, the repercussions of tooth loss in older adults are essential and should be considered in formulating public oral health policies.

Despite the increase in resources for the implantation of regional dental prosthetic laboratories in Brazil [26] prosthetic rehabilitation treatment at public health care services is not yet sufficient to meet the high demand, which may be one of the factors that contributed to the absence of a reduction in the OHIP-14 scores for a large number of older adults evaluated in the present study. Thus, broadening access to dental prosthetic services for older adults could result in a better OHRQoL in this age group [27, 28]. 

OHIP-14 investigates how factors such as weight, sex, and education affect oral health. Emphasizing the connection between oral health and overall well-being, dental caries, inadequate oral hygiene, and aesthetic concerns play a significant role in reducing quality of life. This highlights the essential requirement for effective dental disease prevention programs to enhance individual well-being and counteract these adverse effects. Despite ongoing dental issues causing physical and psychological risks, overcoming obstacles like cost, wait time, and transportation access is crucial to enable people to seek care. Prevention programs for oral diseases, such as OHIP-14, play a critical role in promoting well-being and mitigating the negative impacts associated with dental problems. Addressing barriers like cost, wait time, and transportation access is essential to ensure that individuals can access and benefit from these programs [29, 30].

Critical to avoiding the onset of oral conditions impacting individual psychophysical health is the implementation of targeted prevention programs through proactive oral care. Establishing virtuous and sustainable personal habits requires active health education programs. Despite the vitality of these initiatives, further action is necessary due to the persistence of oral diseases. Essential to preventing the physical, mental, and oral health consequences of dental diseases is combating their widespread problem. This demands a multifaceted approach involving early detection and intervention, improved access to care, and holistic management.

## Conclusion

The present study demonstrated an association of OHRQoL with oral health status variables. The most considerable impact was related to decayed components, missing teeth, tooth pain, and bleeding, which positively correlated with the OHIP-14 domains. These measures have a future in OHRQoL surveys as an adjunct to identify the conditions with the most potential to compromise patient well-being and QoL. It is essential to document the impact of oral health on life quality at any given time to identify the variations in impact among subgroups of the population for planning and evaluating care.

## Data Availability

The datasets used and/or analysed during the current study available from the corresponding author on reasonable request.
